# The S100A8/A9 heterodimer amplifies proinflammatory cytokine production by macrophages via activation of nuclear factor kappa B and p38 mitogen-activated protein kinase in rheumatoid arthritis

**DOI:** 10.1186/ar1939

**Published:** 2006-04-13

**Authors:** Katsue Sunahori, Masahiro Yamamura, Jiro Yamana, Kouji Takasugi, Masanori Kawashima, Hiroshi Yamamoto, Walter J Chazin, Yuichi Nakatani, Satoru Yui, Hirofumi Makino

**Affiliations:** 1 Department of Medicine and Clinical Science, Graduate School of Medicine, Dentistry and Pharmaceutical Sciences, Okayama University, 2-5-1 Shikata-cho, Okayama 700-8558, Japan; 2 Department of Biochemistry and Molecular Vascular Biology, Graduate School of Medical Science, Kanazawa University, 13-1 Takara-machi, Kanazawa 920-8640, Japan; 3 Department of Biochemistry and Physics, Center for Structural Biology, Vanderbilt University, 465 21st Avenue, Nashville, TN 87232-8725, USA; 4 Faculty of Pharmaceutical Sciences, Teikyo University, 1091-1 Sagamiko, Tsukui-gun, Kanagawa 199-0195, Japan

## Abstract

S100A8 and S100A9, two Ca^2+^-binding proteins of the S100 family, are secreted as a heterodimeric complex (S100A8/A9) from neutrophils and monocytes/macrophages. Serum and synovial fluid levels of S100A8, S100A9, and S100A8/A9 were all higher in patients with rheumatoid arthritis (RA) than in patients with osteoarthritis (OA), with the S100A8/A9 heterodimer being prevalent. By two-color immunofluorescence labeling, S100A8/A9 antigens were found to be expressed mainly by infiltrating CD68^+ ^macrophages in RA synovial tissue (ST). Isolated ST cells from patients with RA spontaneously released larger amounts of S100A8/A9 protein than did the cells from patients with OA. S100A8/A9 complexes, as well as S100A9 homodimers, stimulated the production of proinflammatory cytokines, such as tumor necrosis factor alpha, by purified monocytes and *in vitro*-differentiated macrophages. S100A8/A9-mediated cytokine production was suppressed significantly by p38 mitogen-activated protein kinase (MAPK) inhibitors and almost completely by nuclear factor kappa B (NF-κB) inhibitors. NF-κB activation was induced in S100A8/A9-stimulated monocytes, but this activity was not inhibited by p38 MAPK inhibitors. These results indicate that the S100A8/A9 heterodimer, secreted extracellularly from activated tissue macrophages, may amplify proinflammatory cytokine responses through activation of NF-κB and p38 MAPK pathways in RA.

## Introduction

S100A8 and S100A9 are two members of the S100 protein family that are characterized by the presence of two Ca^2+^-binding sites of the EF-hand type. These proteins are also designated as migration inhibitory factor- or myeloid-related protein-8 (MRP8) and MRP14, or calgranulin A and B, respectively [1-3]. Most members of the S100 family exist in the form of homodimers or heterodimers within cells and interact with several effector proteins mostly in a Ca^2+^-dependent manner, thereby regulating enzyme activities, the dynamics of cytoskeleton constituents, cell growth and differentiation, and Ca^2+ ^homeostasis [1]. S100A8 and S100A9 are predominantly expressed in cells of the myelomonocytic lineage; both proteins are present at high concentrations in the cytoplasm of neutrophils and monocytes, and the S100A8/A9 heterodimer is translocated to membrane and cytoskeletal structures upon activation [4-6]. Intracellular S100A8/A9 complexes play an important role in myeloid cell maturation, cell trafficking, and arachidonic acid (AA) metabolism [2].

S100A8 and S100A9 are secreted as complexes from neutrophils and monocytes after activation of protein kinase C in a novel pathway requiring an intact microtubule network [7]. High levels of the proteins have been found in the extracellular milieu during inflammatory conditions such as rheumatoid arthritis (RA) [2,3]. The S100A8/A9 heterodimer, originally identified as an antimicrobial protein, exhibits cytokine-like functions in the local environment, most notably enhancing leukocyte recruitment to inflammatory sites and AA transportation to its target cells [8-10]. However, the nature of surface receptors for S100A8/A9 and its signaling pathways has not yet been fully elucidated. The soluble S100A8/A9 complex binds to the cell surface of endothelial cells by interacting with specific binding sites such as heparin sulfate proteoglycans and novel carboxylated glycans [11,12]. In addition, CD36 and the receptor for advanced glycation endproducts (RAGE) are two other putative receptors for this complex. Interaction of exogeneous S100A8/A9 and AA complexes with the scavenger receptor CD36 facilitates AA uptake by endothelial cells [13]. RAGE, a scavenger receptor belonging to the immunoglobulin (Ig) family that signals to the nuclear factor kappa B (NF-κB) pathway, was identified as a functional receptor for the S100A12 protein [14]. Structural similarities between S100A12 and other S100 proteins [3] and the binding of S100B and S100A12 to RAGE [1] suggest that RAGE may be a general receptor for the S100 family of proteins.

The inflamed synovial membrane in patients with RA is characterized by infiltration of inflammatory cells, primarily lymphocytes, and macrophages and proliferation of synovial fibroblasts, together with increased vascularity. Macrophages play a critical role in the perpetuation of synovial inflammation and joint destruction mainly by secreting proinflammatory cytokines such as tumor necrosis factor alpha (TNF-α) and interleukin 1 (IL-1) [15]. These two cytokines, produced at high levels by macrophages localized in the lining layer and at the pannus lesion, induce the synthesis of numerous inflammatory mediators and matrix-degrading enzymes via the activation of the transcription factor NF-κB and the mitogen-activated protein kinase (MAPK) cascade [15,16].

Many effector molecules are thought to be involved in the TNF-α- and IL-1-driven cascade of proinflammatory events in RA. Concentrations of the S100A8/A9 heterodimer in peripheral blood (PB) in patients with RA have been increased in association with the severity of arthritis [17,18]. More importantly, the protein was more enriched in synovial fluid (SF) than in blood circulation [18], and it was expressed in synovial tissue (ST) macrophages localized in the lining layer adjacent to the cartilage-pannus junction [18,19]. These findings suggest an active role of S100A8/A9 protein in the progressive synovial inflammation, but their functions relevant to RA pathogenesis remain to be determined. In the present study, we confirmed the abundance of S100A8/A9 complexes in the joints of patients with RA and investigated the effects of recombinant S100A8/A9 proteins on monocyte/macrophage cytokine production and activation of NF-κB and MAPK signaling.

## Materials and methods

### Patients and samples

Study patients with RA and control patients with osteoarthritis (OA) were diagnosed according to the revised 1987 criteria of the American College of Rheumatology (formerly, the American Rheumatism Association) [20,21]. All patients with RA were receiving prednisolone at no more than 5 mg/day and various disease-modifying antirheumatic drugs. Paired serum and SF samples were obtained from 17 patients with RA (14 women, 3 men; mean ± standard deviation (SD) age, 63 ± 9 years) and 17 patients with OA (12 women, 5 men; 65 ± 6 years); SF samples were aspirated from the knee joint during therapeutic arthrocentesis. Most patients with RA were active; they had multiple joint tenderness and swelling, systemic inflammatory responses (mean ± SD, serum C-reactive protein [CRP, 53 ± 53 mg/liter] and erythrocyte sedimentation ratio [63 ± 42 mm/hour]), and elevated serum IgM class rheumatoid factor titer (110 ± 133 units/ml), whereas patients with OA did not show evidence of a systemic inflammatory response. ST samples were obtained from patients with RA and patients with OA at the time of total knee joint replacement. PB samples were collected from healthy volunteers. All patients and healthy individuals gave informed consent.

### Isolation and culture of ST cells

Fresh ST samples were fragmented and digested with collagenase and DNase in RPMI 1640 medium (Life Technologies, Gaithersburg, MD, USA) for 1 hour at 37°C. After removal of tissue debris, cells were washed with medium. The resultant single-cell suspensions were adjusted at a density of 1 × 10^6 ^cells per ml in culture medium (RPMI 1640 medium supplemented with 25 mM HEPES, 2 mM L-glutamine, 2% nonessential amino acids, 100 IU/ml penicillin, and 100 μg/ml streptomycin; Life Technologies) with 10% heat-inactivated fetal calf serum (FCS; Life Technologies). The cells were incubated in the wells of six-well plates (Corning, Corning, NY, USA) at 37°C in a humidified atmosphere containing 5% CO_2_. Culture supernatants were harvested 72 hours later and stored at -30°C until the S100A8/A9 assay.

### Immunoassay for S100 proteins and cytokines

Concentrations of S100A8, S100A9, and S100A8/A9 were measured in duplicate by the quantitative sandwich enzyme-linked immunosorbent assay (ELISA) using commercially available kits (BMA Biomedicals AG, Augst, Switzerland) according to the manufacturer's instructions. The detection limits for S100A8, S100A9, and S100A8/9 were 0.69, 0.31, and 4.69 ng/ml, respectively.

Concentrations of TNF-α, IL-1-β, IL-6, IL-8, IL-10, and IL-12 p70 in monocyte culture supernatants were measured by cytometric beads array (CBA) with a series of anticytokine monoclonal antibody (mAb)-coated beads and phycoerythrin-conjugated anticytokine mAbs followed by flow cytometric analysis performed on a FACScan flow cytometer using the CBA kit and CBA software (all three obtained from Becton, Dickinson and Company, San Jose, CA, USA). TNF-α concentrations in some experiments were measured using the ELISA kits (Becton, Dickinson and Company). The detection limits for cytokines were 20 pg/ml.

### Two-color immunofluorescence labeling

Cryostat sections (4 μm) from ST samples were fixed in acetone and blocked with 10% donkey serum for 30 minutes. Double immunofluorescence was performed by serially incubating sections with 1 μg/ml of mouse IgG1 mAb against human S100A8 (8-5C2), S100A9 (S36.48), or S100A8/A9 (27E10, which exclusively recognizes the heterodimer but not the homodimers [22]; BMA Biomedicals AG) or isotype-matched control mAb (BMA Biomedicals AG) at 4°C over night, followed by incubation with rhodamine-conjugated donkey anti-mouse IgG1 mAb (Jackson ImmunoResearch Laboratories, West Grove, PA, USA) for 30 minutes at room temperature, and subsequently with 1 μg/ml of fluorescein isothiocyanate (FITC)-conjugated anti-CD68 mAb (Santa Cruz Biotechnology, Santa Cruz, CA, USA) or control mAb (Santa Cruz Biotechnology) for 30 minutes at room temperature. The double immunofluorescence of sections was examined with an LSM510 inverted laser-scanning confocal microscope (Carl Zeiss, Jena, Germany) and illuminated with 488 and 568 nm of light. Images decorated with FITC and rhodamine were recorded simultaneously through separate optical detectors with a 530-nm band-pass filter and a 590-nm long-pass filter, respectively. Pairs of images were superimposed for colocalization analysis.

### Preparation of S100 proteins

Recombinant human S100A8 and S100A9 proteins were prepared as described previously [23,24]. Briefly, competent *Escherichia coli *strain BL21 (DE3) cells (Novagen, Madison, WI, USA) were transformed with the pET1120-MRP8wt and pET1120 S100A9wt vectors. The transformed cells were grown at 37°C in (2× YT) media supplemented with 100 μg/ml ampicillin for 24 hours; the cells produced the proteins as inclusion bodies. The harvested cells were solubilized with B-PER™ Bacterial Protein Extraction Reagent (Pierce, Rockford, IL, USA). The inclusion bodies were solubilized with Inclusion Body Solubilization Reagent (Pierce), and the proteins were refolded according to the manufacturer's protocol. The proteins were purified by reverse-phase column chromatography (Resource™ RPC; Amersham Bioscience, Buckingamshire, UK) furnished in a BioLogic HR system (Bio-Rad, Hercules, CA, USA), followed by UNO-Q anion exchange chromatography (Bio-Rad). The buffer systems used were the same as those described previously [23]. The purity of the S100A8 protein was found to be greater than 95% by SDS-PAGE (sodium dodecyl sulfate-polyacrylamide gel electophoresis) and silver-staining, followed by densitometric analysis using the ImageJ program (Sun Microsystems, Santa Clara, CA, USA). Because the S100A9 showed weak silver-staining, its purity was estimated by Coomasie Brilliant Blue staining and densitometric analysis to be greater than 95%. The endotoxin content was measured using an endotoxin-specific assay kit (Endospecy; Seikagaku Kogyo, Tokyo, Japan), and the 10 μM solutions of recombinant S100A8 and S100A9 used in the present study contained endotoxin at 0.255 and 0.467 ng/ml, respectively. The mixture of equal amounts of S100A8 and S100A9 solutions was found by its specific ELISA to mostly form the heterodimer.

### Stimulation of monocytes and *in vitro*-differentiated macrophages with S100 proteins

PB mononuclear cells (PBMCs) were prepared from heparinized PB samples of healthy individuals by Ficoll-Hypaque density gradient centrifugation. Monocytes were purified from PBMCs by negative selection using monocyte isolation kit II (Miltenyi Biotec, Bergisch Gladbach, Germany) according to the manufacturer's instructions. The monocyte suspensions were adjusted at a density of 1 × 10^6 ^cells per ml in culture medium with 10% FCS and were incubated in the wells of 48-well plates (Corning) with or without 0.1–10 μM of S100A8, S100A9, and S100A8/A9 in the presence of 100 μg/ml polymyxin B sulfate (ICN Pharmaceuticals, Costa Mesa, CA, USA). Culture supernatants were harvested 24 hours later and stored at -30°C until the cytokine assay. Because the S100 preparations contained as much as 0.722 ng of endotoxin, we determined whether TNF-α induction in monocytes stimulated by 1.0 ng/ml lipopolysaccharide (LPS; Sigma, St. Louis, MO, USA) was blocked by 100 μg/ml polymyxin B.

Monocytes were incubated at a density of 1 × 10^6 ^cells per ml in culture medium with 10% FCS for 7 days with or without 10 ng/ml granulocyte-macrophage colony-stimulating factor (GM-CSF) or 1 ng/ml interferon gamma (IFN-γ). The *in vitro*-differentiated macrophages were stimulated for 24 hours with 2 μM S100A8/A9 in the presence of polymyxin B, and culture supernatants were measured for TNF-α concentrations.

### Preparation of endogenous secretory RAGE

Recombinant human endogenous secretory RAGE (esRAGE) was prepared as described previously [25]. Briefly, COS-7 cells stably were transformed with pCI-*neo *(Promega, Madison, WI, USA) carrying the esRAGE cDNA. The esRAGE protein was purified from conditioned media of the transfectant with an AKTA purifier system using HiTrap-Heparin column, RESOURCE S column, and HiTrap desalting column (Amersham Bioscience), sequentially. The purified material largely consisted of the 50-kDa esRAGE, as evidenced by immunoreactivity with the antibody against the peptide unique to the C truncated-type RAGE [25].

### Effects of RAGE and CD36 blockade on S100A8/A9 stimulation

To examine the possibility of CD36 or RAGE as a putative receptor for S100A8/A9 on monocytes, the monocyte suspensions, at a density of 1 × 10^6 ^cells per ml in culture medium with 10% FCS in 48-well plates, were stimulated for 24 hours by 10 μM S100A8/A9 with or without 2% vol/vol goat anti-RAGE polyclonal Ab (Chemicon International, Temecula, CA, USA) or control goat serum (Chemicon International), 25 μg/ml of esRAGE or human serum albumin (Sigma), and 20 μg/ml of mouse IgG2a/κ anti-CD36 mAb (NeoMarkers, Fremont, CA, USA) or mouse isotype-matched control mAb (NeoMarkers) in the presence of polymyxin B. Culture supernatants were measured for cytokine concentrations.

### Effects of MAPK inhibitors and NF-κB inhibitors on S100A8/A9 stimulation

To examine the involvement of MAPK and NF-κB activation in S100A8/A9-stimulated monocyte cytokine production, monocytes, at a density of 1 × 10^6 ^cells per ml in culture medium with 10% FCS in 48-well plates, were preincubated with 1 μM of the cell-permeable MAPK inhibitors PD98059, SB202190, and SB203580 and the negative control SB202474 (Calbiochem, San Diego, CA, USA), or with or without 1 μM of the inhibitors of NF-κB activation, 1-pyrrolidinecarbodithioic acid (PDTC; Calbiochem), N^α^-tosyl-phenylalanylchloromethylketone (TPCK; Affiniti Research Products, Mamhead Castle, UK), and BAY 11-7082 (Calbiochem) for 2 hours and were then stimulated for 24 hours with or without 0.1–10 μM S100A8/A9 in the presence of polymyxin B. Culture supernatants were measured for cytokine concentrations.

### Detection of p38 MAPK activation

To detect the activation of p38 MAPK by S100A8/A9, monocytes, at a density of 1 × 10^6 ^cells per ml in culture medium with 10% FCS in 48-well plates, were stimulated for 30 minutes with 10 μM S100A8/A9 and were immediately placed in the lysing buffer (Passive Lysis Buffer; Promega). The activation of p38 MAPK in cell lysates was determined using ELISA kits for p38 MAPK protein and p38 MAPK protein phosphorylated on threonine 180/tyrosine 182 (pThr^180^/pTyr^182^) (Sigma) according to the manufacturer's instructions.

### Nuclear extract and electrophoretic mobility shift assay for NF-κB

To examine the effects of S100A8/A9 on NF-κB activation, monocytes, at a density of 1 × 10^6 ^cells per ml in culture medium with 1% FCS in polypropylene tubes (Becton, Dickinson and Company), were stimulated with or without 0.1–10 μM S100A8/A9 in the presence of polymyxin B. Cells were collected 30 minutes later, and nuclear proteins were prepared using a nuclear extract kit (Active Motif, Carlsbad, CA, USA) according to the manufacturer's instructions. In the experiments of p38 MAPK inhibition, monocytes were preincubated with the MAPK inhibitors (PD98059, SB202190, and SB203580) and the control SB202474, then stimulated for 30 minutes by 10 μM S100A8/A9, and nuclear proteins were prepared. The nuclear protein content was measured by the Lowry method using a DC protein assay kit (Bio-Rad). Electrophoretic mobility shift assay (EMSA) was performed using the Nushift NF-κB p65 kit (Active Motif) according to the manufacturer's instructions. Nuclear extracts were incubated with [α-^32^P] dATP (Amersham, Little Chalfont, UK)-labeled double-stranded oligonucleotide probe in binding buffer for 20 minutes. Samples and positive controls (nuclear proteins prepared from monocytes stimulated with 100 ng/ml LPS) were electrophoresed on 5% polyacrylamide gel, followed by autoradiography. To verify the specificity of NF-κB protein binding, competition and supershift analysis was performed by adding an excess of unlabeled competitor or mutant oligonucleotides and rabbit polyclonal anti-NF-κB p65 Ab (Active Motif) to the incubation on ice for 20 minutes before the binding reaction.

### Statistical analysis

Samples with values below the detection limit for the assay were regarded as negative and assigned a value of 0. Data were expressed as the mean value ± standard error of the number of samples evaluated. The statistical significance of differences between two groups was determined by the Mann-Whitney *U *test or the Wilcoxon signed rank test; *p *values less than 0.05 were considered significant. The correlation coefficient was obtained by the Spearman rank correlation test.

## Results

### Increased concentrations of the S100A8/A9 heterodimer in serum and SF from patients with RA

**Figure 1:**
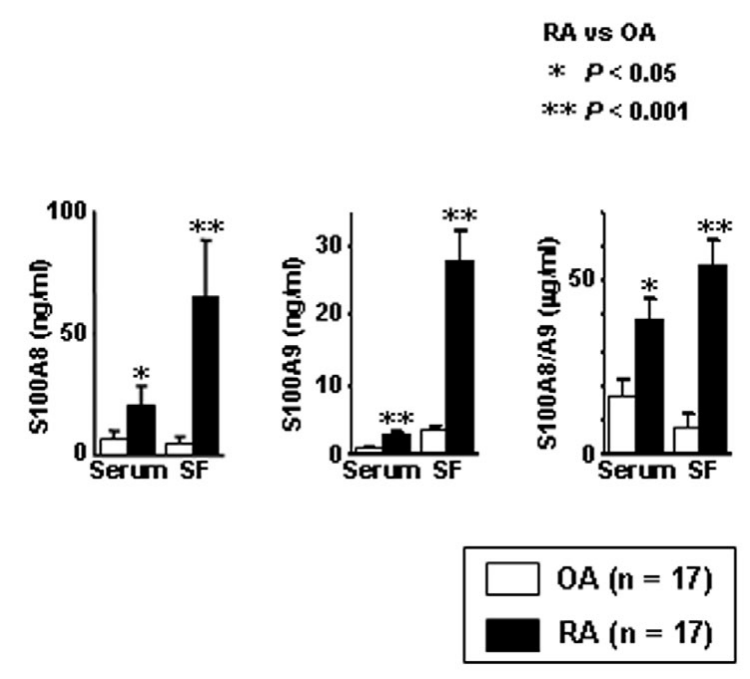
Concentrations of S100A8 and S100A9 proteins in the serum and synovial fluid (SF) of rheumatoid arthritis (RA) and osteoarthritis (OA). Paired serum and SF samples were prepared from patients with RA and patients with OA. Concentrations of the S100A8 and S100A9 homodimer and the S100A8/A9 heterodimer were measured in duplicate by enzyme-linked immunosorbent assay. Values are the mean ± standard error of the mean. *n*, number of samples tested.

Concentrations of S100A8, S100A9, and the S100A8/A9 heterodimer in paired serum and SF samples obtained from 17 patients with RA and 17 patients with OA were compared by ELISA. The levels of S100A8, S100A9, and S100A8/A9 in serum and SF were all significantly increased in patients with RA (20.4 ± 7.8 ng/ml, 2.9 ± 0.3 ng/ml, and 38.9 ± 6.0 mg/ml in serum and 65.3 ± 23.4 ng/ml, 27.9 ± 4.5 ng/ml, and 54.8 ± 7.2 μg/ml in SF, respectively) as compared with patients with OA (7.0 ± 3.0 ng/ml, 0.9 ± 0.1 ng/ml, and 16.8 ± 4.8 μg/ml in serum and 5.1 ± 2.2 ng/ml, 3.6 ± 0.4 ng/ml, and 7.3 ± 4.5 μg/ml in SF, respectively) (Figure 1). The amounts of S100A8/A9 heterodimers detected by ELISA were approximately 1,000-fold greater than those of S100A8 and S100A9 homodimers. S100A8/A9 was present at higher concentrations in SF than in serum in patients with RA, but not in patients with OA, and the serum levels correlated positively with serum CRP levels in patients with RA (*r *= 0.802, *p *< 0.0001). These results suggest that S100A8 and S100A9 proteins may be secreted mainly as the heterodimer from inflammatory cell infiltrates, such as neutrophils and monocytes/macrophages, in the joints of patients with active RA; however, these proteins are thought to be secreted also as the homodimer, because the expression pattern of S100A8 and S100A9 has been shown to differ *in vivo*, depending on cell types, cell differentiation, and inflammatory conditions [6,22,26].

### High-level expression of S100A8/A9 by CD68^+ ^macrophages in RA ST

**Figure 2:**
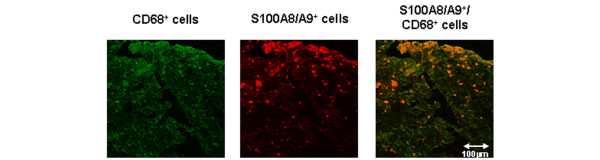
Expression of CD68 and the S100A8/A9 heterodimer in the synovial tissue (ST) of rheumatoid arthritis (RA). Cryostat sections of ST samples from patients with RA were incubated with mouse immunoglobulin (Ig) G1 monoclonal antibody (mAb) against the S100A8/A9 complex or control mAb, followed by incubation with rhodamine-conjugated donkey anti-mouse IgG1 mAb, and were then incubated with fluorescein isothiocyanate-conjugated anti-CD68 mAb. Two-color immunofluorescence confocal images were obtained for CD68 and S100A8/A9 expression (green and red staining, respectively). The two images were superimposed; double-positive cells are shown in yellow. No isotype-matched control mAb was observed. Similar staining patterns were obtained in additional analyses with two synovial tissue samples from different patients.

S100A8/A9 proteins are expressed by infiltrating tissue macrophages during inflammation but not by resident tissue macrophages [2,3]. To determine S100A8/A9 expression at the site of macrophage infiltration, the proliferative ST samples from three patients with RA were analyzed by two-color immunofluorescence labeling with anti-S100A8/A9 Ab and anti-CD68 Ab. Figure 2 shows representative staining patterns of the S100A8/A9 protein and the CD68 antigen in the ST. These tissues were characterized by the extensive infiltration of CD68-expressing macrophages. Colocalization analysis revealed that the heterodimeric S100A8/A9 was highly expressed by CD68^+ ^macrophages, in particular the cells localized to the lining layer. However, S100A8/A9 staining was negligible in CD68-negative cells in the sublining layer, including lymphocytes and vascular endothelial cells. Immunostaining of the S100A8 and S100A9 homodimers shows similar staining patterns, but the intensity and number of S100A8 and S100A9 staining were less prominent (data not shown). These results indicate that S100A8 and S100A9 are expressed predominantly in the form of a heterodimeric complex by highly activated macrophages in RA ST.

### S100A8/A9 secretion *in vitro *by RA ST cells

**Figure 3:**
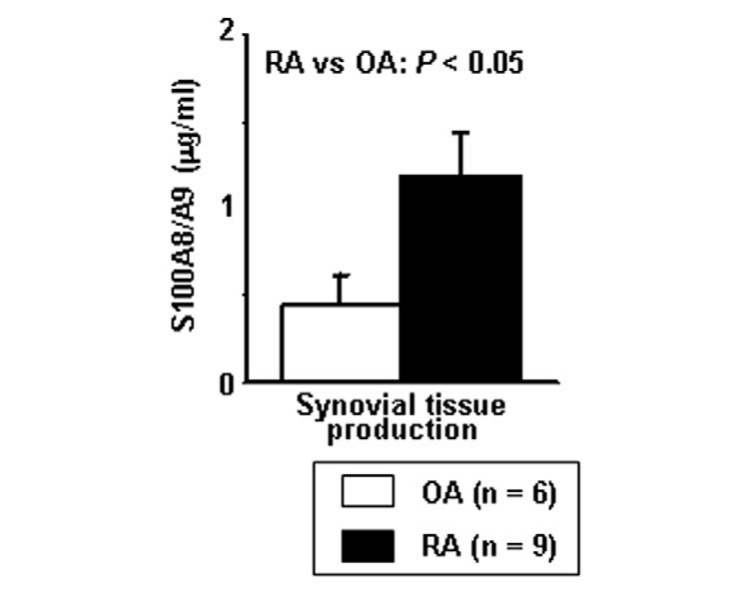
Secretion of S100A8 and S100A9 proteins from isolated synovial tissue (ST) cells of rheumatoid arthritis (RA) and osteoarthritis (OA). Freshly isolated ST cells (1 × 10^6 ^cells per ml in culture medium with 10% fetal calf serum) were incubated without any stimulation for 72 hours. Culture supernatants were measured in duplicate for the S100A8/A9 heterodimer by enzyme-linked immunosorbent assay. Values are the mean ± standard error of the mean. *n*, number of samples tested.

To confirm the local production of S100A8/A9 proteins at the site of chronic inflammation in RA, isolated cells from ST samples of nine patients with RA and six patients with OA were incubated for 72 hours without any stimulation and culture supernatants were measured for the heterodimer by ELISA. As shown in Figure 3, ST cells from patients with RA spontaneously secreted larger amounts of S100A8/A9 proteins (1.20 ± 0.25 μg/ml) than did the cells from patients with OA (0.45 ± 0.17 μg/ml). Lactate dehydrogenase activity in these culture supernatants was undetectable or detectable only at negligible levels, indicating that S100A8/A9 is released as a consequence of active secretion but not of cellular injury.

Although the cellular composition of ST cells was not determined in this study, we had previously measured the frequencies of nonspecific esterase-staining macrophages and CD3-positive T cells in the ST cell population isolated in the same manner and found that RA ST samples consisted of 31.7 ± 5.3% (mean ± SD) macrophages and 44.7 ± 6.6% T cells (*n *= 7) and that OA ST samples consisted of 30.3 ± 4.9% macrophages and 28.8 ± 3.3% T cells (*n *= 4) (unpublished data). Additionally, we could not detect significant neutrophil contamination (≤1%) in two of the studied ST samples. Therefore, it would seem that the increased S100A8/A9 secretion from RA ST cells may be due to the presence of *in vivo*-activated macrophages in the culture. This notion is likely consistent with the fact that tissue macrophages are more activated in RA than in OA [15,16].

### Proinflammatory cytokine production by monocytes and *in vitro*-differentiated macrophages after S100A8/A9 stimulation

Purified blood monocytes were stimulated for 24 hours by diluted concentrations (0.1–10 μM) of recombinant S100A8 and S100A9 proteins in the presence of 100 μg/ml polymyxin B, and culture supernatants were measured for cytokine concentrations by immunoassay. Polymyxin B was able to effectively block TNF-α production by 1 ng/ml LPS (1,259 ± 125 pg/ml versus 36 ± 4 pg/ml; *n *= 3), but TNF-α production by 10 μM S100A8/A9 decreased from 1,874 ± 51 pg/ml to 955 ± 40 pg/ml with polymyxin B. The results indicate that endotoxin contamination in the S100 proteins was not completely neutralized by polymyxin B and that low levels of endotoxin act in synergy with S100 proteins.

**Figure 4:**
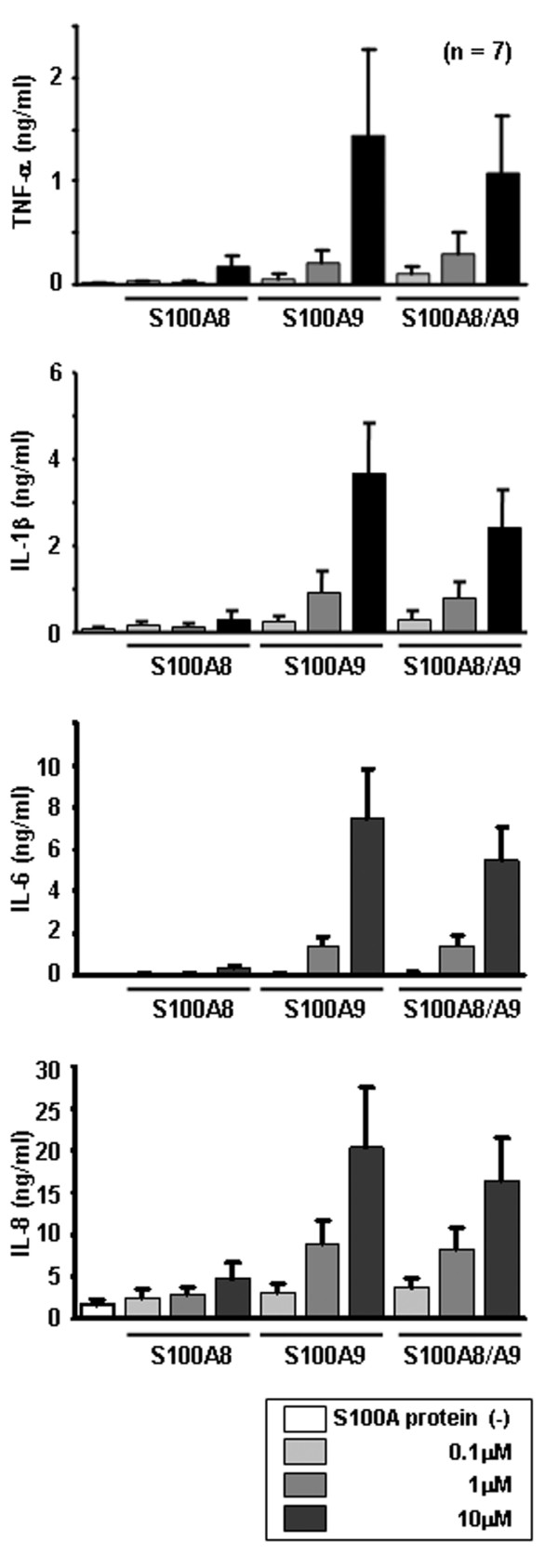
Induction of proinflammatory cytokine production by monocytes after stimulation with S100A8, S100A9, and S100A8/9. Monocytes were purified by negative selection from peripheral blood mononuclear cells of healthy individuals. The monocyte suspensions (1 × 10^6 ^cells per ml in culture medium with 10% fetal calf serum) were incubated with or without S100A8, S100A9, and S100A8/A9 (0.1–10 μM) in the presence of polymyxin B (100 μg/ml). Culture supernatants were harvested 24 hours later. Cytokine concentrations were measured by cytometric beads array (using anticytokine monoclonal antibody [mAb]-coated beads and phycoerythrin-conjugated anticytokine mAbs). Values are the mean ± standard error of the mean. IL, interleukin; *n*, number of samples tested; TNF-α, tumor necrosis factor alpha.

As shown in Figure 4, S100A9 and S100A8/A9 induced the production of TNF-α, IL-1-β, IL-6, and IL-8, but not of IL-10 and IL-12 p70, in a dose-dependent manner; all values for the complex were lower than those for the A9 homodimer, although not statistically significant. In contrast, S100A8 failed to induce significant levels of cytokine production. It is thus likely that the S100A9 subunit in the heterodimer is critical in activating the proinflammatory signaling.

**Figure 5:**
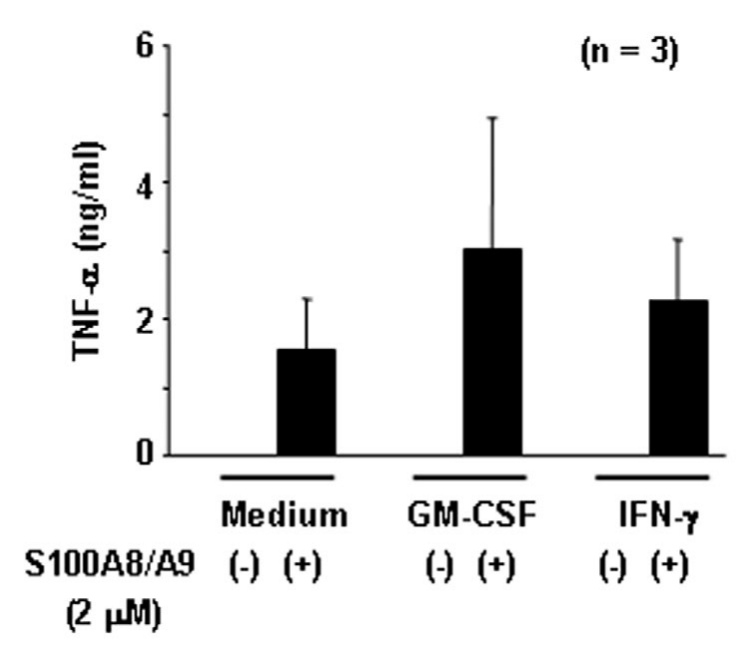
Induction of tumor necrosis factor alpha (TNF-α) production by *in vitro*-differentiated macrophages after S100A8/A9 stimulation. Monocytes (1 × 10^6 ^cells per ml in culture medium with 10% fetal calf serum) were incubated for 7 days with or without 10 ng/ml granulocyte-macrophage colony-stimulating factor (GM-CSF) or 1 ng/ml interferon gamma (IFN-γ). The *in vitro*-differentiated macrophages were then stimulated for 24 hours with 2 μM S100A8/A9 in the presence of polymyxin B (100 μg/ml). Culture supernatants were measured for TNF-α concentrations by enzyme-linked immunosorbent assay. Values are the mean ± standard error of the mean. *n*, number of samples tested.

Next, *in vitro*-differentiated macrophages, prepared by incubating monocytes for 7 days with or without GM-CSF or IFN-γ, were stimulated for 24 hours by 2 μM S100A8/A9 (50 μg/ml; equivalent to approximately the mean concentration in RA SF), and TNF-α production was determined. As shown in Figure 5, these macrophages, particularly when treated with cytokines, could respond well to S100A8/A9 by producing TNF-α. These results suggest that the secreted S100A8/A9 heterodimer may amplify the local expression of proinflammatory cytokines in joints of patients with RA.

### Effects of RAGE and CD36 blockade on S100A8/A9 stimulation

To examine whether S100A8/A9 used RAGE and CD36 as signal transducing receptors on monocytes, monocytes were stimulated for 24 hours by 10 μM S100A8/A9 with or without anti-RAGE Ab, esRAGE (the splice variant lacking the transmembrane-spanning domain), and anti-CD36 Ab, and culture supernatants were measured for cytokine concentrations by immunoassay. The S100A8/A9-stimulated cytokine response was not significantly reduced by blockade of the RAGE or CD36 receptor (percentage of inhibition, < 30%). Therefore, neither RAGE nor CD36 appears to be relevant to S100A8/A9 stimulation in monocytes.

### Involvement of p38 MAPK activation in S100A8/A9 stimulation

**Figure 6:**
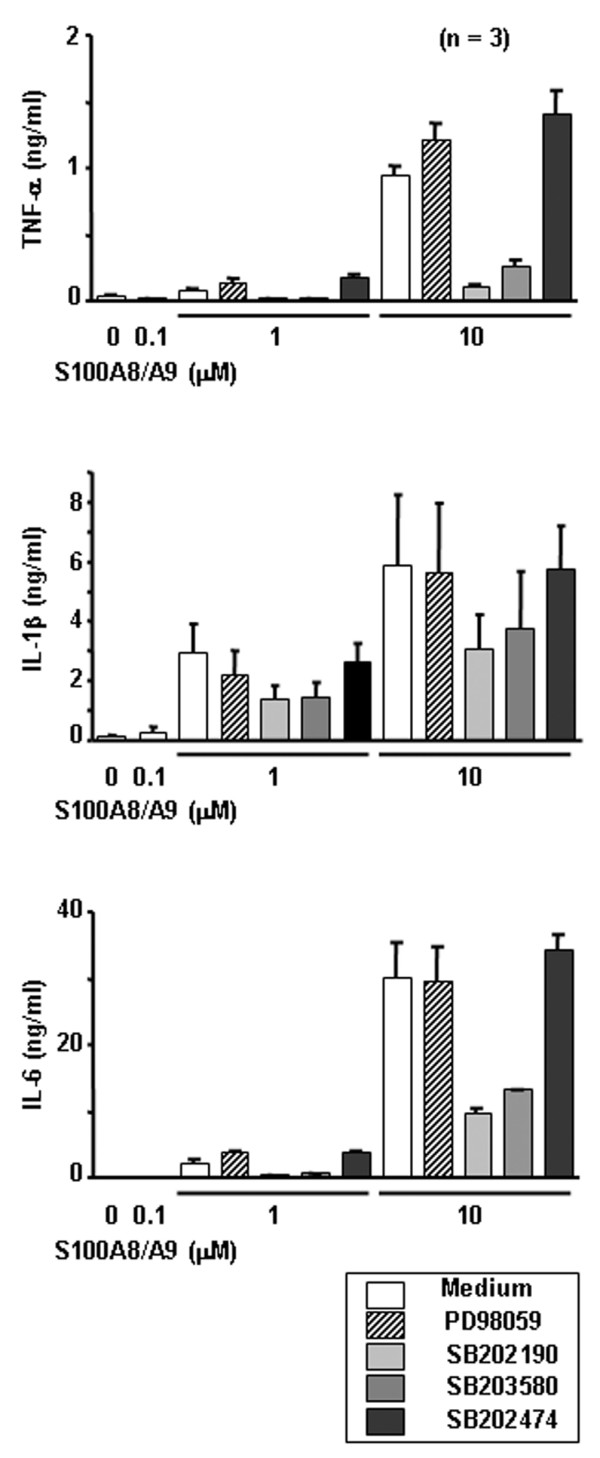
Effects of mitogen-activated protein kinase (MAPK) inhibitors on S100A8/A9-mediated cytokine production. Monocytes from healthy individuals (1 × 10^6 ^cells per ml in culture medium with 10% fetal calf serum) were preincubated for 2 hours in the presence of the MAPK inhibitors PD98059, SB202190, and SB203580 and the negative control SB202474 (1 μM) and were then stimulated for 24 hours with or without S100A8/A9 (0.1–10 μM) in the presence of polymyxin B (100 μg/ml). Culture supernatants were measured for cytokines by cytometric beads array. Values are the mean ± standard error of the mean. IL, interleukin; *n*, number of samples tested; TNF-α, tumor necrosis factor alpha.

To examine whether MAPK activation was involved in the S100A8/A9-mediated cytokine response, monocytes were pretreated with the MAPK inhibitors (PD98059, SB202190, and SB203580) and the control SB202474 and were then stimulated for 24 hours by 1–10 μM S100A8/A9. Culture supernatants were measured for cytokine concentrations by immunoassay. As shown in Figure 6, TNF-α, IL-1-β, and IL-6 production by S100A8/A9-stimulated monocytes was clearly reduced by the specific p38 MAPK inhibitors SB202190 and SB203580, while it was not affected by the MEK (mitogen-activated/extracellular signal-regulated kinase) inhibitor PD98059, as well as by SB202474.

To support the significance of p38 MAPK in S100A8/A9 stimulation, monocytes were stimulated for 30 minutes by 10 μM S100A8/A9, and p38 MAPK protein and phosphorylated p38 MAPK protein (pThr^180^/pTyr^182^) in cell lysates were measured by ELISA. The phosphorylation ratio of p38 MAPK protein (phosphorylated p38 MAPK protein/total p38 MAPK protein) was markedly higher in S100A8/A9-stimulated monocytes (2.17 ± 0.74 U/pg; *n *= 3) than in unstimulated monocytes (0.52 ± 0.09 U/pg), proving the activation of p38 MAPK by S100A8/A9.

### Involvement of NF-κB activation in S100A8/A9 stimulation

**Figure 7:**
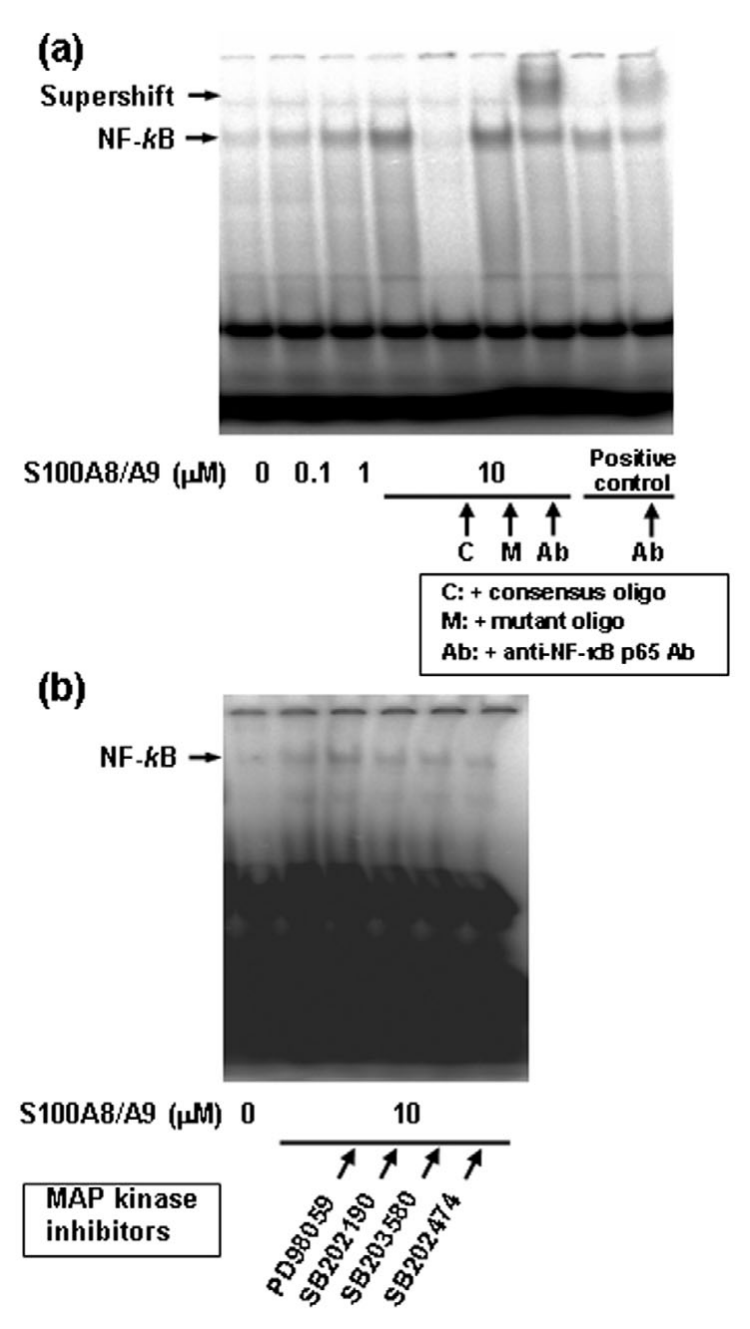
S100A8/A9-induced nuclear factor kappa B (NF-κB) activation and its independence of p38 mitogen-activated protein kinase (MAPK) activation. **(a) **Activation of the transcriptional factor NF-κB in S100A8/A9-stimulated monocytes. Monocytes (1 × 10^6 ^cells per ml in culture medium with 1% fetal calf serum) were stimulated for 30 minutes with or without S100A8/A9 (0.1–10 μM) in the presence of polymyxin B (100 μg/ml). Nuclear proteins were extracted from the cells, and electophoretic mobility shift assay (EMSA) was performed, as described in Materials and methods. Nuclear proteins prepared from monocytes stimulated with 100 ng/ml of lipopolysaccharide were used as positive controls. The specificity of NF-κB protein binding was verified by inhibition and supershift experiments with unlabeled NF-κB consensus or mutant oligonucleotides and anti-NF-κB p65 antibody. **(b) **Effects of p38 MAPK inhibition on S100A8/A9-induced NF-κB activation. Monocytes, after two-hour pretreatment with or without the MAPK inhibitors (PD98059, SB202190, and SB203580) and the control compound SB202474, were stimulated for 30 minutes with or without S100A8/A9 (10 μM), and nuclear proteins were prepared. The DNA-binding activity of NF-κB was determined by EMSA.

**Figure 8:**
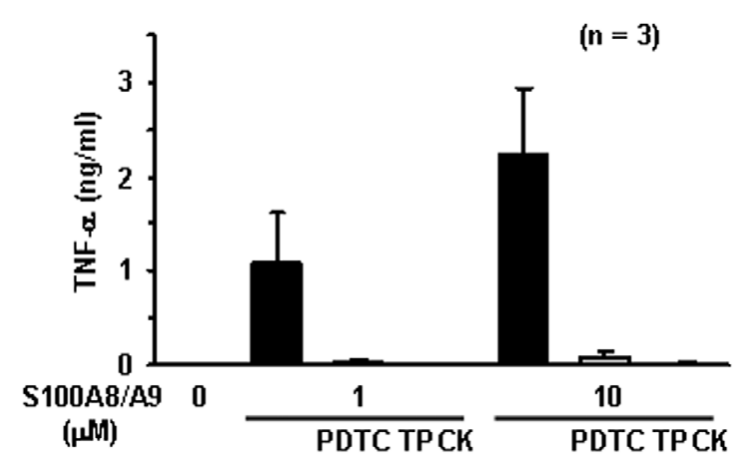
Effects of nuclear factor kappa B (NF-κB) inhibitors on S100A8/A9-mediated cytokine production. Monocytes (1 × 10^6 ^cells per ml in culture medium with 10% fetal calf serum), after two-hour pretreatment with or without the inhibitors of NF-κB activation (1 μM), 1-pyrrolidinecarbodithioic acid (PDTC), and N^α^-tosyl-phenylalanylchloromethylketone (TPCK), were stimulated for 24 hours with or without S100A8/A9 (1 or 10 μM) in the presence of polymyxin B. Culture supernatants were measured for tumor necrosis factor alpha (TNF-α) concentrations by enzyme-linked immunosorbent assay. Values are the mean ± standard error of the mean. *n*, number of samples tested.

The transcription factor NF-κB plays a prominent role in the activation of multiple inflammatory genes in RA [15,16]. To determine the activation of NF-κB in S100A8/A9-stimulated monocytes, monocytes were stimulated for 30 minutes with or without 0.1–10 μM S100A8/A9 and nuclear proteins were measured for the DNA-binding activity of NF-κB by EMSA. As shown in Figure 7a, the NF-κB activity was induced by S100A8/A9 in a dose-dependent manner, verifying the activation of NF-κB by S100A8/A9. To further determine the significance of NF-κB activation in S100A8/A9 stimulation, monocytes were pretreated with or without 1 μM of the NF-κB inhibitors PDTC and TPCK and then stimulated for 24 hours by 1 or 10 μM S100A8/A9, and culture supernatants were measured for TNF-α concentrations. As shown in Figure 8, TNF-α production by S100A8/A9-stimulated monocytes was almost completely reduced by PDTC and TRCK. Similar results were also obtained with the selective NF-κB pathway inhibitor BAY 11-7082 (data not shown). Therefore, NF-κB activation may be essential for the transcriptional activation of many cytokine genes in monocytes stimulated by S100A8/A9.

To explore the interaction of p38 MAPK with NF-κB activation in S100A8/A9 stimulation, monocytes, pretreated with or without the MAPK inhibitors and the control compound, were stimulated for 30 minutes by 10 μM S100A8/A9 and nuclear proteins were measured for NF-κB activation by EMSA. As shown in Figure 7b, MAPK inhibitors did not significantly reduce the S100A8/A9-induced NF-κB activity, which was verified by analysis of the images using NIH Image (National Institutes of Health, Bethesda, MD, USA). Thus, these two signaling pathways are likely to be independently involved in cytokine production by S100A8/A9-stimulated monocytes, and post-transcriptional and/or translational mechanisms may be important in the p38 MAPK pathway.

## Discussion

The expression of both S100A8 and S100A9, two members of the S100 family of Ca^2+^-binding proteins, in infiltrating tissue macrophages has been associated with chronic inflammatory conditions such as RA [2]. In the present study, we found that the S100A8/A9 heterodimer is expressed predominantly by CD68^+ ^macrophages in the ST of patients with RA. In addition, using the recombinant proteins, we demonstrated that heterodimeric S100A8/A9, as well as homodimeric S100A9, stimulates monocytes and *in vitro*-differentiated macrophage to produce proinflammatory cytokines such as TNF-α. S100A8/A9 stimulation is reduced significantly by p38 MAPK inhibitors and NF-κB inhibitors and induces the activation of the transcription factor NF-κB. Therefore, S100A8/A9 is considered to amplify proinflammatoy cytokine responses through activation of NF-κB and p38 MAPK pathways in RA.

The S100A8/A9 heterodimer has been shown to be a reliable indicator of disease activity and joint inflammation in inflammatory rheumatic diseases, including RA [18,19], juvenile RA (JRA) [27-30], psoriatic arthritis [18], and spondylarthropathy [18]. Consistent with this finding, both serum and SF levels of S100A8/A9 were significantly higher in patients with RA than in patients with OA, with a positive correlation between serum S100A8/A9 and CRP levels. S100A8 and S100A9 are independently expressed in some pathological conditions [26], but both proteins were increased predominantly in the form of a heterodimeric complex in the joints of patients with active RA. S100A8 and S100A9 molecules preferentially assemble to noncovalently associated complexes in a Ca^2+^-dependent manner [31], and heterodimers are secreted extracellularly from neutrophils, monocytes, and tissue macrophages, but not from lymphocytes, under inflammatory conditions [4,5,7].

The concentration of S100A8/A9 in neutrophils constitutes up to 60% of total cytosolic protein [2], and an abundance of S100A8/A9 complexes can be thus readily released from neutrophils during activation and cell death. In RA, neutrophils are localized mainly to the SF, but not to the synovial membrane, and act as prominent effectors of inflammation and cartilage damage by releasing various enzymes [32]. The relationship of S100A8/A9 levels in SF to the degree of local neutrophil infiltration was demonstrated in inflammatory arthritides, including RA [18], suggesting the active secretion from infiltrating neutrophils. On the other hand, S100A8 and S100A9 are secreted from monocytes as heterodimeric complexes after activation of protein kinase, which is induced by different inflammatory stimuli [7]. We found that S100A8/A9 was intensively expressed by CD68^+ ^macrophages in RA ST and that isolated RA ST cells spontaneously secreted higher levels of the protein than did OA ST cells. Taken together, these findings indicate that infiltration and activation of neutrophils and macrophages may be responsible for the elevated concentration of S100A8/A9 in joints of patients with RA.

Our immunofluorescence studies showed the distribution of S100A8/A9-expressing macrophages in both the lining and sublining layer of RA ST, mostly corresponding with earlier studies of the expression pattern in the ST from RA [5,18,19], JRA [28], and psoriatic arthritis [18]. Monocytes expressing cell surface S100A8/A9 represent a fast-migrating monocyte subpopulation across the endothelium barrier, and high levels of the S100A8/A9 protein are secreted after their interaction with inflammatory-activated endothelial cells [28,33]. S100A8/A9 expression by sublining macrophages, particularly by perivascular macrophages thought to represent newly infiltrated macrophages, is attributable to selective infiltration of S100A8/A9^+ ^monocytes and their endothelium-dependent induction of S100A8/A9 proteins, because the inflamed tissue of RA is characterized by increased vascularity and endothelial cell activation [34]. On the other hand, S100A8 mRNA expression in macrophages has been shown to be stimulated by cytokines such as TNF-α, IFN-γ, and IL-10 [35,36], which are relevant to RA pathogenesis [16]. Thus, cytokine stimulation may be responsible for S100A8/A9 expression by synovial lining macrophages (that is, the highly activated phenotype of tissue macrophages that are capable of producing high levels of cytokines such as TNF-α) [15]. It is intriguing to note that there is a positive correlation between S100A8/A9 expression and TNF-α release in alveolar macrophages from patients with active sarcoidosis [37].

The released S100A8/A9 heterodimer may play a role in the propagation of inflammation by recruiting neutrophils and monocytes to joints of patients with RA. Extracellular S100A8/A9 enhances the transendothelial migration of these inflammatory cells by enhancing CD11b expression and affinity for intracellular adhesion molecule-1 (ICAM-1) [33,38]. We found that S100A8/A9 stimulated monocytes and *in vitro*-differentiated macrophages to produce proinflammatory cytokines such as TNF-α. In addition, co-stimulation of S100A8/A9 with LPS and cytokines such as TNF-α, IL-1-β, and IFN-γ showed synergistic effects on monocyte cytokine production (data not shown). However, S100A8/A9 was less effective in terms of monocyte stimulation compared with cytokines; the protein of 10^-7 ^to 10^-5 ^M concentrations (2.4–240 μg/ml) was required for significant cytokine induction, whereas cytokines are known to produce their actions in the range of 10^-12 ^to 10^-9 ^M concentrations [39]. Despite its lower efficacy, S100A8/A9 stimulation is believed to be effective in joints of patients with RA, where the protein is present at 54.8 ± 28.6 μg/ml, because *in vitro*-differentiated macrophages, as well as freshly isolated monocytes (data not shown), when stimulated with 2 μM (50 μg/ml) S100A8/A9, could produce significant levels of TNF-α. Similarly, 15 μg/ml S100A8/A9 significantly induced IL-8 release from bronchial epithelial cells, and anti-S100A8/A9 Ab treatment reduced the IL-8-stimulating potential of bronchial secretions [40]. More recently, the study using microarray analysis demonstrated that S100A8/A9 proteins (100 μg/ml) directly activate endothelial cells to induce proinflammatory chemokines and adhesion molecules and to increase vascular permeability [41]. Taken together, these findings suggest the significance of S100A8/A9 as key effectors/amplifiers of inflammation with a wide range of activities, including cytokine induction.

Considering the potential inflammatory properties, there seems to be a discrepancy between high concentrations of serum S100A8/A9 and the lack of its peripheral effect in patients with RA. We speculate that the yet-unidentified, inhibitory molecules for S100A8/A9 activity might be present in the blood circulation, as is well recognized in the regulation of TNF-α and IL-1 activities by soluble TNF receptors and IL-1 receptor antagonist which bind to the protein or interfering with its cell interaction, respectively [15,16]. However, it is also conceivable that an abundance of the protein might partially contribute to monocyte activation before entry into the joint, as evidenced by the increase of circulating CD16^+ ^mature monocytes in patients with RA [42].

The S100A9 homodimer, but not the S100A8 homodimer, stimulated the monocyte/macrophage cytokine response. Similarly, the S100A9 homodimer, as well as the S100A8/A9 complex, could enhance ICAM-1 binding to monocytes, whereas the S100A8 homodimer failed to do so [33]. The S100A9 subunit is thus likely to be critical in eliciting the inflammatory signaling pathway. Interestingly, values of cytokine production with S100A8/A9 tend to be lower than those with S100A9, which may indicate partial inhibition of S100A9 stimulation by its heterodimeric partner S100A8. However, we could not clearly detect inhibitory effects of S100A8 on S100A9-induced TNF-α production when titrating S100A8 concentrations (0.1–10 μM) into the 1 μM S100A9 solution (data not shown), although the possibility still remains that much higher concentrations of S100A8 are required for its significant inhibition. The S100A9 protein stimulates the β_2 _integrin (CD11/CD18)-mediated neutrophil adhesion by inducing the high-affinity CD11b epitope, and this S100A9 activity is specifically inhibited by S100A8 [38]. More importantly, recent studies have demonstrated that numbers of S100A8/A9-expressing macrophages increased in the ST of patients with RA after treatment with high dose of intravenous methylprednisolone and that high levels of S100A8 induced by glucocorticoids have anti-inflammatory properties independent of hetero-complex formation with S100A9 [43]. These findings support the possible inhibition of the heterodimer-induced cytokine response by the S100A8 subunit.

RAGE, originally defined by its binding to advanced glycation endproducts, has been identified as a multiligand receptor that can bind inflammatory molecules such as HMGB1 (high mobility group box chromosomal protein 1), S100A12, and S100B1 [14,44]. Significant similarities in structure among S100A8, S100A9, and S100A12 suggest that RAGE may function as a signal-transducing receptor for these S100 proteins. In addition, the CD36 molecule, a member of the scavenger receptor family, has been shown to bind the complex of S100A8/A9 and AA and induce uptake of exogenous AA into the endothelial cells [13]. However, S100A8/A9-mediated cytokine production was not inhibited by anti-RAGE Ab, esRAGE (an endogenous inhibitor of RAGE activity), or anti-CD36, excluding the possibility of RAGE or CD36 as a putative receptor for S100A8/A9. Thus, S100A8/A9 may induce cell activation after its nonspecific interaction with monocytes, because S100A8/A9 binds endothelial cells in conjunction with heparin sulfate proteoglycans and novel carboxylated glycans [11,12].

The significance of NF-κB and MAPK pathways in the inflammatory gene activation in joints of patients with RA has been well established [15,16]. We found that S100A8/A9, like TNF-α and IL-1, efficiently activate both the NF-κB and p38 MAPK pathways, resulting in cytokine production in monocytes.

Because MAPK inhibitors did not reduce the DNA-binding activity of NF-κB, these two critical signaling pathways may be independently involved in S100A8/A9-mediated cytokine production. NF-κB inhibitors could completely block the cytokine response. Therefore, we believe that the transcriptional activity of NF-κB may be most critical in S100A8/A9-induced cytokine expression in monocytes/macrophages and that p38 MAPK may regulate the synthesis of cytokines at the post-transcriptional and/or translational levels

## Conclusion

In summary, the S100A8/A9 heterodimer, highly expressed by synovial lining macrophage, may play a role in amplifying proinflammatory cytokine responses via activation of NF-κB and p38 MAPK in RA.

## Abbreviations

AA = arachidonic acid; CBA = cytometric beads array; CRP = C-reactive protein; ELISA = enzyme-linked immunosorbent assay; EMSA = electrophoretic mobility shift assay; esRAGE = endogenous secretory receptor for advanced glycation endproducts; FCS = fetal calf serum; FITC = fluorescein isothiocyanate; GM-CSF = granulocyte-macrophage colony-stimulating factor; ICAM-1 = intracellular adhesion molecule-1; IFN-γ = interferon gamma; Ig = immunoglobulin; IL = interleukin; JRA = juvenile rheumatoid arthritis; LPS = lipopolysaccharide; mAb = monoclonal antibody; MAPK = mitogen-activated protein kinase; MRP = myeloid-related protein, NF-κB = nuclear factor kappa B; OA = osteoarthritis; PB = peripheral blood; PBMC = peripheral blood mononuclear cell; PDTC = 1-pyrrolidinecarbodithioic acid; PE = phycoerythrin; RA = rheumatoid arthritis; RAGE = receptor for advanced glycation endproducts; SD = standard deviation; SF = synovial fluid; ST = synovial tissue; TNF-α = tumor necrosis factor alpha; TPCK = N^α^-tosyl-phenylalanylchloromethylketone.

## Competing interests

The authors declare that they have no competing interests.

## Authors' contributions

KS was responsible for the experiments and data analysis and wrote the report. MY was responsible for the planning of the research and wrote up the manuscript. JY, KT, and MK assisted the experiments. HY prepared recombinant esRAGE, and WJC, YN, and SY prepared recombinant S100A8 and S9 proteins. HM critically read the manuscript. All authors read and approved the final manuscript.
